# Successful re-establishment of a rabbit population from embryos vitrified 15 years ago: The importance of biobanks in livestock conservation

**DOI:** 10.1371/journal.pone.0199234

**Published:** 2018-06-18

**Authors:** Francisco Marco-Jiménez, Manuel Baselga, José Salvador Vicente

**Affiliations:** Institute of Science and Animal Technology, Laboratorio de Biotecnología de la Reproducción, Universidad Politécnica de Valencia, Valencia, Spain; Embrapa, BRAZIL

## Abstract

Genetic resource banks (GRB) are a valuable tool for maintaining genetic variability and preserving breeds from pathogens or catastrophe, enabling us to assess and correct breeding schemes, minimizing the impact of genetic drift and facilitating diffusion. This study tests their efficiency in re-establishing two extinct populations of a synthetic rabbit line selected for daily weight gain, using vitrified embryos from two generations (18th and 36th) separated by 15 years of genetic selection. The effect of long-term storage of vitrified embryos in liquid nitrogen was also evaluated. A total of 516 vitrified embryos using the same protocol were transferred into 54 recipients. The embryos had been maintained in liquid nitrogen during 2 different periods, (i) 1 year (301 embryos and 26 transfers, 36th generation) and (ii) 15 years (259 embryos and 28 transfers, 18th generation). A total of 80.0% (8/10 to 18th) and 60.0% (9/15 to 36th) of the foundational sire families were eventually re-established. Over approximately one year, animals within each population were crossed to produce the next generation and re-establish the original population size. Our study demonstrated that our GRB of embryos vitrified 15 years ago is a successful strategy to re-establish rabbit populations to continue the breeding programme.

## Introduction

The Animal Science Department set up a genetic resource bank (GRB) in 1993 to assist the Universidad Politécnica de Valencia (UPV) in the development and management of a genetic improvement programme for meat rabbits to meet the need for animals required by rabbit meat producers. Intensive meat production in rabbits is based on a three-way crossbreeding scheme involving maternal and paternal synthetic lines to produce the animals for consumption [1]. The rabbit breeding industry is increasingly using selected lines [2]. Work on developing synthetic lines in Spain began at the UPV in 1976. Every two or three generations of selection, embryos from a representative sample of the matings (for each male, sire families) are vitrified and stored. This is a typical example of ex situ in vitro conservation to safeguard genetic resources against disasters [3]. Embryos have the great advantages of ensuring the conservation of a breed’s whole genome and the speed with which breeds can be reconstructed [4]. In rabbits, the efficiency of embryonic vitrification to produce offspring ranged between 25–65%, depending on the genetic breed and whether the embryo transfer was from a single donor or a pool of different donors [2, 5–11]. Our embryo bank contains more than 11,000 embryos from four maternal lines (A, V, H and LP) and one paternal line (R). Recently, line A reached generation 45, 40 in line V, 10 in line LP and 37 in line R. These lines have been kept closed at the same selection nucleus and subjected to the same selection and management programme since their foundation [12]. Generating and characterizing these lines requires great effort and they must be kept in stock even if they are out of any commercial use [13].

The broadest definition of a GRB refers to a storage facility for gametes and embryos from threatened populations, with the specific and deliberate aim of using them in a breeding programme on some future occasion. Among the criteria for setting up a cryobank are: (i) offspring will be rederived from the cryobank; (ii) rederived offspring will have exhibited the desired genotype; and (iii) the rederived offspring should produce offspring [14]. GRBs have been described as a valuable tool for maintaining genetic variability or preserving selected lines from pathogens or catastrophe, and allow us to evaluate genetic improvement, minimizing the impact of genetic drift and facilitating the diffusion of the lines to different countries [2, 14–17]. In essence, GRBs are “repositories” offering the possibility of recreating breeds or breeding lines in case they are lost. Some examples of the re-establishment of populations have been described in polytocous species such as mice, rats, and rabbits [18,19]. Nonetheless, one of the most recurrent issues was related to how long cryopreserved embryos can be stored for [20]. In recent years, relatively limited published data have shown that long-term cryopreservation had no adverse effect on their post-thaw survival, implantation rates, clinical pregnancy, miscarriage and live birth (up to 20 years in humans [21]; up to 3 years in pigs [22]; after 15 years in bovine [23]; after 13 years in sheep [24]; and after 15 years in rabbit [25]). However, these results have not been replicated in all reports on the topic. Testart et al. [26] reported that only several months of cryopreservation decreased the pregnancy rate of human embryos. In addition, Mozdarani & Moradi [27] showed that long-term cryopreservation reduced viability and increased chromosome aberrations in murine embryos. Besides, the influence of cryopreservation techniques on stability across storage time is unknown. Has been suggested that stability of vitrified embryos might be more vulnerable to environmental factors such as pressure or temperature due to alterations in the liquid nitrogen level, which could cause cracking or fracturing in the glassy state [25, 28].

Against this background, the aim of the present study was to test the efficiency of using our rabbit embryo cryobank to re-establish populations and the effect of long-term storage in liquid nitrogen of embryos vitrified 15 years ago.

## Materials and methods

All chemicals, unless otherwise stated, were reagent-grade and purchased from Sigma-Aldrich Química S.A. (Alcobendas, Madrid, Spain). All the experimental procedures used in this study were performed in accordance with Directive 2010/63/EU EEC for animal experiments and reviewed and approved by the Ethical Committee for Experimentation with Animals of the Universidad Politécnica de Valencia, Spain (research code: 2015/VSC/PEA/00061).

### Animals

**Fig 1:**
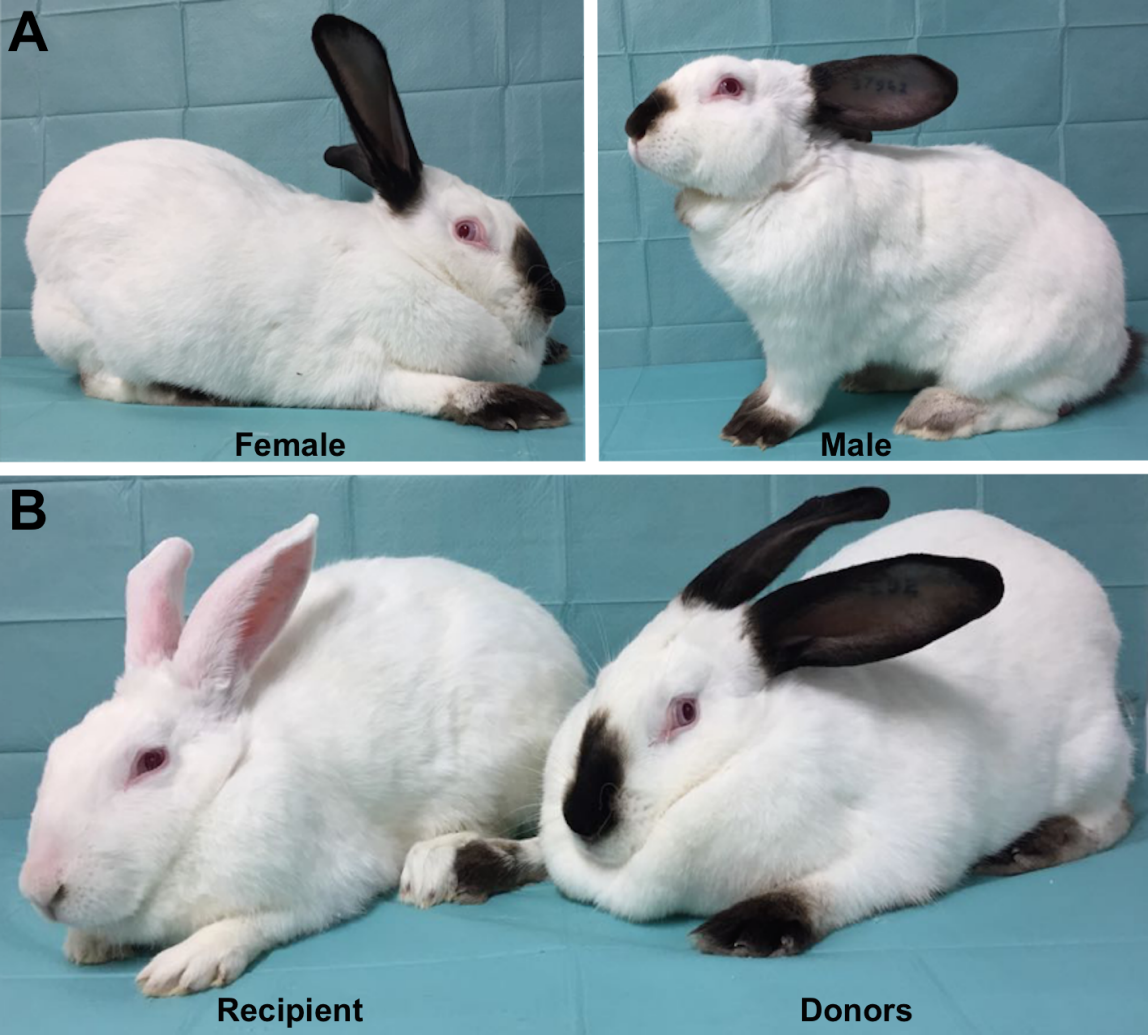
Animals used in this study belonged to 2 Spanish commercial rabbit lines. (A) Female and male from synthetic line R selected on individual daily weight gain between weaning (day 28) and end of the fattening (day 63) over 37 generations (B). Females used as recipient from maternal line based on New Zealand White rabbits selected since 1980 by a family index for litter size at weaning during 45 generations and donor from synthetic line R.

The experiment was carried out with animals from two Spanish commercial rabbit lines (designated Line R and Line A, Fig 1) reared at the Universitat Politécnica de Valencia. Line R comes from the fusion of two paternal lines, one founded in 1976 with Californian rabbits reared by Valencian farmers and another founded in 1981 with rabbits belonging to specialized paternal lines [29]. The selection method was individual selection on post-weaning daily gain, with weaning taking place at 28 days and the end of the fattening at 63 days. The current generation of selection is the 36th. The size of this line is around 120 does and 25 males. This number of males is enough for breeding with keeping the inbreeding coefficient at low level. For the same reason, matings between relatives sharing a grandparent were avoided, and each male contributed at least one offspring to the next generation. Selection was conducted in non-overlapping generations and the generation interval was around 10 months. Young rabbits were weaned at 28-days-old and the fattening period lasted 5 weeks. The first mating took place when the rabbits were around 4.5-months-old, and after kindling the new mating was tried 21 days later. All lines had been kept closed since their foundation. Line A is based on New Zealand White rabbits selected since 1980 by a family index for litter size at weaning over 45 generations [30].

All animals were housed at the Universitat Politécnica de Valencia experimental farm in flat deck indoor cages (75×50×30 cm), with free access to water and commercial pelleted diets (minimum of 15 g of crude protein per kg of dry matter (DM), 15 g of crude fibre per kg of DM, and 10.2 MJ of digestible energy (DE) per kg of DM). The photoperiod was set to provide 16 h of light and 8 h of dark, and the room temperature was regulated to keep temperatures between 14°C and 28°C.

### In vivo embryo production and collection

**Fig 2:**
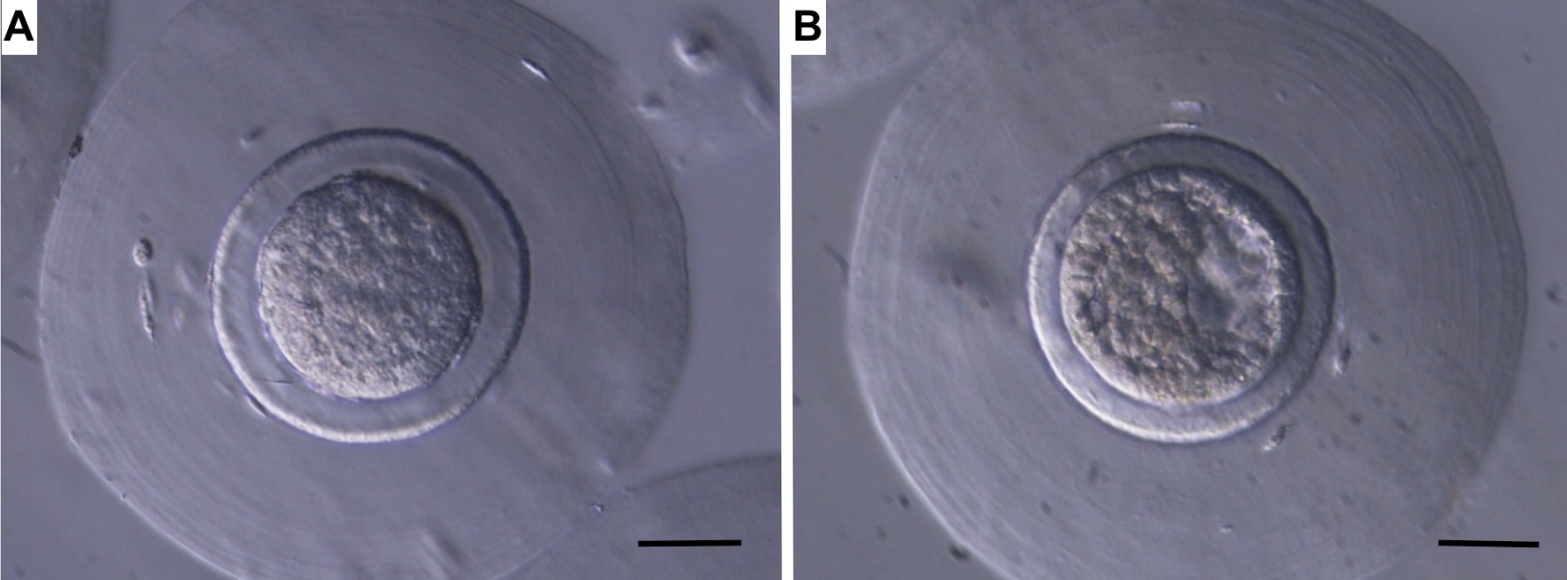
Rabbit embryos classified as excellent or good (presenting homogenous cellular mass, mucin coat, and spherical zona pellucida according to International Embryo Transfer Society classification). (A) Compacted morulae at 100x. (B) Early blastocyst at 100x. Scale bar: 50 μm.

All embryos were from line R, while females from line A were used solely as recipients. Embryos were obtained from females after the third birth. To this end, females were inseminated with semen from an unrelated male to avoid an increase in consanguinity. In addition, males were selected within sire families in order to reduce inbreeding. Another practice to reduce inbreeding was the avoidance of mating between animals having common grandparents. At the time of artificial insemination, females were administered 1 μg of buserelin acetate (Hoechst Marion Roussel S.A., Madrid, Spain) to induce ovulation and euthanized 72 hours later with an intravenous injection of 200 mg/Kg of pentobarbital sodium (Dolethal, Vétoquinol, Madrid, Spain). Then, embryos were recovered by perfusion of each oviduct and uterine horn with 10 mL pre-warmed Dulbecco Phosphate Buffered Saline (DPBS) supplemented with 0.2% of Bovine Serum Albumin (BSA). After recovery (compacted morulae and early blastocysts, Fig 2), only embryos classified as excellent or good (presenting homogenous cellular mass, mucin coat, and spherical zona pellucida according to International Embryo Transfer Society classification) were vitrified.

### Vitrification and warming procedure

The vitrification procedure is described in detail elsewhere [7]. Briefly, the vitrification was carried out in two steps at room temperature (approx. 20°C–22°C). In the first step, embryos from each donor doe were placed for 2 minutes in an equilibrium solution consisting of 12.5% dimethyl sulphoxide (DMSO) and 12.5% of ethylene glycol (EG) in DPBS supplemented with 0.1% of BSA. In the second step, embryos were suspended for 1 minute in the vitrification solution containing 20% DMSO and 20% EG in DPBS supplemented with 0.1% of BSA. Then, embryos suspended in vitrification medium were loaded into 0.125 ml plastic straws (ministraws, L'Aigle, France) and plunged directly into liquid nitrogen. After storage in liquid nitrogen, embryos were warmed by horizontally placing the ministraw 10 cm from liquid nitrogen for 20 to 30 seconds; when the crystallization process began, the straw was submerged into a water bath at 20°C for 10 to 15 seconds. The vitrification solution was removed while loading the embryos into a solution containing DPBS and 0.33 M sucrose for 5 minutes, followed by one bath in a solution of DPBS for another 5 minutes before transfer.

### Embryo transfer by laparoscopy

Immediately after warming, embryos were evaluated morphologically, and only embryos without damage in mucin coat or pellucid zone were transferred into the oviduct (unilateral transfer) of pseudopregnant recipient females from line A following the procedure described by Besenfelder and Brem [31]. Ovulation was induced with an intramuscular dose of 1 mg of Buserelin Acetate (Suprefact, Hoechst Marion Roussel S.A, Madrid, Spain) 68–72 hours before transfer.

Briefly, the equipment used was a Karl Storz laparoscope, which is a 0°-mm straight-viewing laparoscope, 30-cm in length, with a 5-mm working channel (Karl Storz Endoscopia Ibérica S.A. Madrid, Spain). Recipients were sedated by intramuscular injection of 5 mg/Kg of xylazine (Bayer AG, Leverkusen, Germany). As surgical preparation for laparoscopy, anaesthesia was performed by intravenous injection into the marginal ear vein of 6 mg/Kg of ketamine hydrochloride (Imalgene®, Merial, S.A., Lyon, France). During laparoscopy, 12 mg of morphine hydrochloride (Morfina®, B. Braun, Barcelona, Spain) was administered intramuscularly. First, the abdominal region was shaved, and the animals were then placed on an operating table in a vertical position (head down at 45-degree angle). This vertical positioning ensures that the stomach and intestines are cranially located so that the Fallopian tubes form a downwardly pointing loop between the ovaries and uterus. Only an endoscope trocar was inserted into the abdominal cavity. When the trocar was removed, the abdomen was insufflated with CO2 and the endoscope was then inserted. For embryo transfer, embryos were aspirated in a 17-gaugue epidural catheter (Vygon corporate, Paterna, Valencia, Spain), introduced into the inguinal region with an epidural needle and then inserted in the oviduct through the infundibulum. After transfer, does were treated with antibiotics (4mg/Kg of gentamicin every 24h for 3 days, 10% Ganadexil, Invesa, Barcelona, Spain) and analgesics (0.03mg/Kg of buprenorphine hydrochloride, [Buprex®, Esteve, Barcelona, Spain] every 12 hours for 3 days and 0.2mg/Kg of meloxicam [Metacam® 5mg/mL, Norvet, Barcelona, Spain] every 24h for 3 days).

### Effect of population on implantation rate, offspring rate at birth and embryonic and foetal losses

Implantation rates were assessed by laparoscopy following the previous procedure, noting the number of embryos implanted at day 12 from total embryos transferred and birth rate (offspring at birth/total embryos transferred expressed as percentage). Embryonic losses were calculated as the difference between transferred embryos and implanted embryos expressed as percentage. Foetal losses were calculated as the difference between total offspring at birth and implanted embryos expressed as percentage.

### Evaluation of viable population size

The numbers of individuals for both generations were determined at birth, at weaning (28-days-old) and at adult age (5-months-old, sexual maturity). Finally, for one year approximately, founders within each generation were crossed to produce offspring and then re-establish the original population size. The total numbers of individuals for both populations were determined at the end of the fattening at 63 days (selection age).

### Experimental design

**Fig 3:**
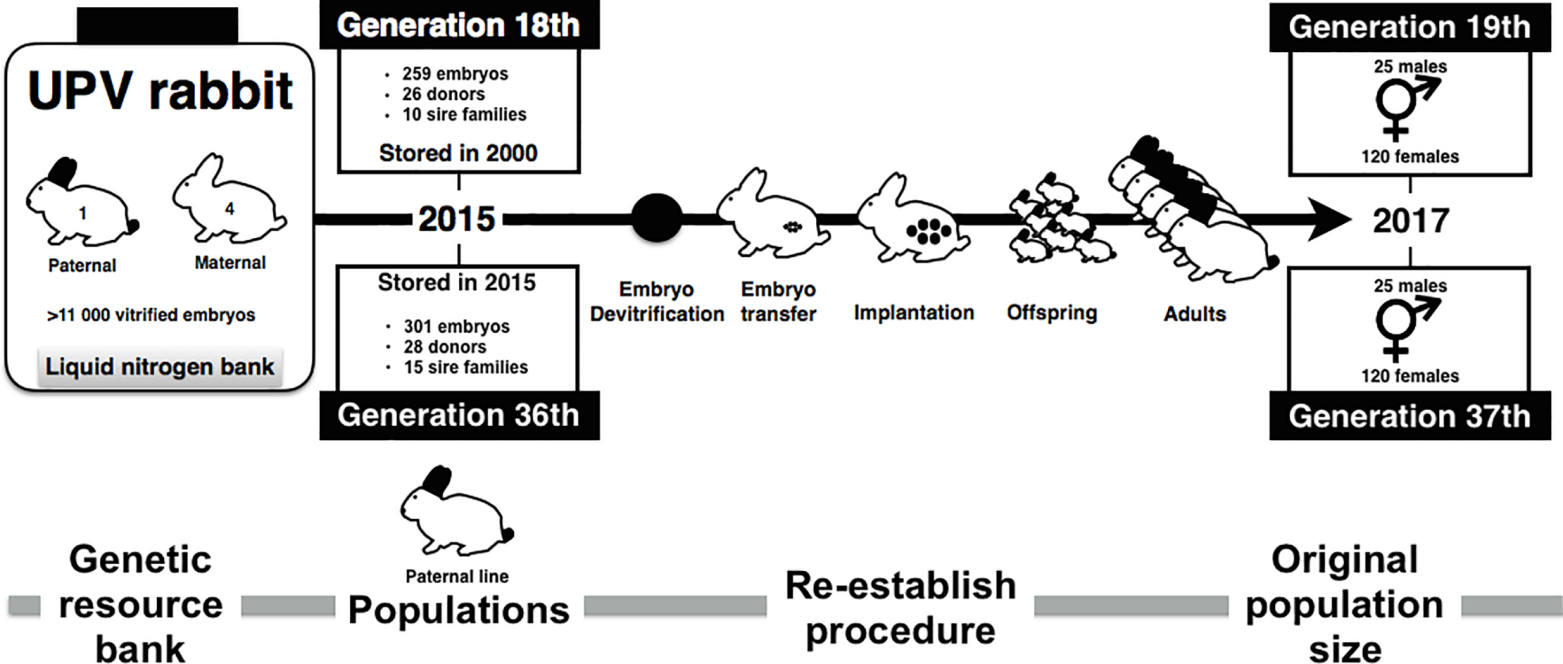
Experimental scheme design.

The experimental design of this study is shown in Fig 3. A total of 560 embryos stored in liquid nitrogen before re-establishment were disbanded (259 stored for up 15 years, belonging to the 18th generation of selection, and 301 stored for 1 year, belonging to the 36th generation of selection). After thawing, a total of 516 were transferred to 54 recipients. The mean number of transferred embryos per recipient doe was 9.6 (ranged from 6 to 15) for the 18th generation and 10.5 (ranged from 6 to 19) for the 36th generation. The embryo transfers were performed in 9 sessions. Note that the same operator, in the same place, performed the vitrification procedure for both populations, so there is no operator effect in this experiment. A total of 28 embryo donors from 10 sire families belonged to the 18th generation, while 26 embryo donors from 15 sire families belonged to the 36th generation. Rederived offspring were crossed to produce the original population size with 120 females and 25 males. Matings between relatives sharing a grandparent were avoided.

### Statistical analysis

The effects of storage on the percentage of embryos retrieved from the ministraw after warming and the percentage of transferable embryos were compared using a general linear model including the storage time in liquid nitrogen with two levels (15 and 1 year) as a fixed factor. The error was designated as having a binomial distribution using probit link function. Binomial data for implantation rate, offspring rates at birth (total and alive) and embryonic and foetal loss rates were assigned as 1 if positive development had been achieved, or a 0 if it had not. To compare litter size, a general linear model was also performed, including the storage time in liquid nitrogen with two levels (15 and 1 year) as a fixed factor as previously.

A P value of less than 0.05 was considered to indicate a statistically significant difference. The data are presented as least square mean ± standard error mean. All statistical analyses were carried out using a commercially available software program (SPSS 21.0 software package; SPSS Inc., Chicago, Illinois, USA, 2002).

## Results

### Transferable embryo after warming

Table 1 shows the results on the overall rate of retrieved embryos after warming and the judged transferable embryo rate for both cryostorage periods. There were no differences between storage period for all the variables studied. A total of 251 (94±1.5%) and 287 (90±1.7%) embryos were retrieved after warming (P = 0.086), of which 244 (66±5.6%) and 272 (79±4.0%) were judged transferable (P = 0.106), for embryos stored for up 15 and 1 years, respectively.

**Table 1:** Influence of storage period on transferable embryo rate from 2 extinct populations of a commercial rabbit line separated by 15 years of genetic selection.

Cryostorage period (years)	Generation	Number of embryos		
		Vitrified (%)	Retrieved (%)	Transferable* (%)
15	18th	259 (100±0.0)	251 (94±1.5)	244 (66±5.6)
1	36th	301 (100±0.0)	287 (90±1.7)	272 (79±4.0)

* Embryos without damage in mucin coat or zona pellucida after warming

### Pregnancy and birth rate and progeny mature sexually

After transfer, there were no significant differences in implantation rate (36±3.0% vs. 38±2.8%, P = 0.699, Table 2), offspring at birth rate (total: 22±2.6% vs. 23±2.4%, P = 0.796 and alive: 18±2.4% vs. 22±2.4%, P = 0.306, Table 2) and losses rate (embryonic: 64±3.0% vs. 62±2.8%, P = 0.699 and foetal: 39±5.0% vs. 39±4.6%, P = 0.987, Table 2) between both populations (embryos stored for up to 15 years and 1 year). Likewise, there were no significant differences in litter size (3.5±0.60 vs. 4.6±0.65, for embryos stored for up 15 and 1 years, respectively, P = 0.260).

**Table 2:** Cryopreservation efficiency from two extinct populations of a commercial rabbit line separated by 15 years of genetic selection.

Cryostorage period (years)	N			Implantation rate (%)	Offspring at birth rate (%)	Losses rate (%)		Alive born offspring	Weaned^c^	*Mature sexually Age*^d^
		Recipient does	Pregnancy rate (%)			Embryonic^a^	Fetal^b^			
15 (18^th^ generation)	244	26	50.0	36±3.0	22±2.6	64±3.0	39±5.0	41	35	17
1 (36^th^ generation)	272	28	53.6	38±2.8	23±2.4	62±2.8	39±4.6	69	60	28

N: number of transferred embryos.

^a^ Calculated as differences between transferred embryos and implanted embryos expressed as percentage.

^b^ Calculated as differences between implanted embryos and offspring at birth expressed as percentage. Data are presented as least squares means ± standard error of the least squares means.

^c^ Weaned at 28-days-old.

^d^ Mature sexually age at 5 months.

Of the 28 recipients used for embryos stored for up 15 years, 15 had 41 offspring with at least one male from 8 different sire families and 15 females, while of the 26 recipients used for embryos stored for up 1 year, 13 had 69 offspring, with at least one male from 15 different sire families and 26 females. A total of 35 and 60 offspring were weaned at 28-days-old for embryos stored for up 15 and 1 years, respectively. Of these, 17 and 28 reached sexual maturity (5 months) for embryos stored for up 15 and 1 years, thus generating the founders within population for the 18th and 36th generation.

### Re-establishment of the original population size

Founders within each population, 12 females and 5 males for the 18th generation and 18 females and 10 males for the 36th generation were crossed. A total of 166 litters were produced over a year (81 and 85 for the 18th and 36th generation, respectively, Table 3) and a total of 629 offspring were obtained (247 and 382 for the 18th and 36th generation, respectively, Table 3). Of these, 378 offspring were weaned at 28-days-old (144 and 234 for the 18th and 36th generation, respectively, Table 3). Finally, 347 reached selection age (137 and 210 for the 18th and 36th generation, respectively, Table 3).

**Table 3:** Re-establishment of the original population sizes for both cryostorage periods.

Cryostorage period (years)	Founders				Offspring		
	Sire families	Females	Males	Parities	Total born	Weaned^a^	Selection age^b^
15 [18^th^ generation]	8	12	5	81	247	144	137
1 [36^th^ generation]	9	18	10	85	382	234	210

^**a**^ Weaned at 28-days-old.

^b^ Selection age at 63-days-old (selection age)

## Discussion

Little is known about the efficiency of using GRBs to re-establish mammal populations, particularly with livestock embryos, as cryopreservation has generally been applied as a tool for the storage and exchange of valuable animals. Embryo cryopreservation is widely used to re-establish breeding colonies in laboratory animals [32]. Our current study demonstrates that after 15 years, vitrified embryos continued to maintain the same capacity to regenerate a rabbit population. The relevance of this study becomes higher considering that our goal was the preservation of the whole genome to ensure reproduction, population integrity and heterozygosity to continue with the genetic selection programme after re-establishment. Besides, the present study rules out confounding factors such as cryopreservation procedure, operator and environment, as the experiment was carried out by the same operator, in the same environment and using the same cryopreservation procedure. Altogether, our data show that using ex situ in vitro conservation strategies to cryopreserve animal genetic resource banks is a valuable tool to guarantee genetic diversity in rabbit.

At present, millions of offspring have been born from cryopreserved embryos of more than 40 mammalian species [33]. Nevertheless, embryo cryopreservation is only routinely performed in cattle, while in other domestic animal species its application is practically reduced to experimental use [20]. In contrast, this study tests the efficiency of our GRB following long-term embryo storage to re-establish populations, which is a totally different scenario. In this context, it is important to recall that the criteria for establishing a GRB are that offspring will be rederived from the cryobank, rederived offspring shall exhibit the desired genotype and the rederived offspring must produce offspring [14]. Our results demonstrated that all of these criteria were met. In both populations, sufficient numbers of offspring were obtained after thawing and transfer to re-establish foundation populations. Besides, these results showed that long-term storage of vitrified embryos in liquid nitrogen maintains pregnancy rate, fertility and offspring rate, in accordance with previous results [21–25].

Moreover, in this study, GRB efficiency based on the total number of offspring regenerated by the number of thawed embryos was 23.0%. This has been posited as the only measure that accurately reveals real-world production of offspring from cryopreserved embryos [14]. These data are comparable to those reported by our laboratory in 2003 using the same donor and recipient genotypes, vitrification procedure and operator [18]. Although the efficiency may seem somewhat low in comparison with the current state-of-the-art in terms of offspring in rabbits (ranged between 25–65% [2, 5–11]), the origin of the embryos (pool of embryos which come from various donor does) and number of transferred embryos (fixed) of these studies differed greatly, which may have been behind the differences. In our study, the transferred embryos belonged to the same donor doe, which is crucial from a genetic point of view to avoid inbreeding problems [18]. Furthermore, the efficiency also may be due in part to the genotype used in this study. Several reports have shown that donor genotype or another factor such as recipient genotype significantly influences the cryopreservation outcome [11, 14, 34]. Although Line R is genetically able to grow faster, females presented severe reproductive problems such as implantation embryo failure and lower litter size, related with higher gestational and foetal losses [35–36].

Collectively, the data indicate that a "minimal bank" of 259 embryos was sufficient to re-establish the foundation population for this genotype. However, genetic diversity has been identified as an important factor influencing a population's long-term potential for survival [37]. In rabbit, from one population to another, their number should be around 120 embryos if the goal is the preservation of a particular allele at a given locus and 330 embryos from 15 males and 30 females if the goal is the conservation of genetic combinations involving several loci. Interestingly, and in line with previous data [38], we obtained a large enough number of sire families for both populations (8 and 9 for generation 18th and 36th respectively) to continue to assess the genetic gain of the selection process. In rabbit, it has been shown that an effective preservation of characteristics such as growth rate could be obtained with the offspring of 9 sire families for heritability of 0.25 and variation coefficient of 0.1036 [18]. With these results, it will be possible to guarantee an inbreeding coefficient value of less than 1% per generation.

In this case, we applied the vitrification technique with the commonly used ministraw device, developed 23 years ago in our laboratory [6]. Although the latest approach to improving the vitrification procedure is based on small volumes to provide extremely high cooling and warming rates [39–42], in rabbit only slight benefits have been observed when Cryotop® was used with late embryos in terms of offspring [10–11]. Besides, the use of these minimum volume essential devices to establish a GRBs presents some limitations due to the expensive cost and the low number of embryos that they can hold, which is a major drawback for routine embryo cryopreservation in polytocous animals [20]. In addition, the ministraw device minimizes the chances of pathogen transmission during storage exchange and rederivation [43–45] and can be easily labelled using commercial printers before embryo packaging and cryopreservation for easy identification [46]. Embryos cryopreserved in closed systems are essential for the maintenance, relocation and rederivation of populations [20].

Our results here clearly demonstrate that vitrified-warmed embryos stored in liquid nitrogen for 15 years maintained the same capacity to regenerate a disbanded population. Indeed, the theoretical discussion on the duration of storage is one of the most recurring issues in cryobiology [20]. In this sense, the development of embryos from cryopreserved embryos with different storage times does not appear to have any negative effects on pregnancy outcome in several species of mammals [21–25]. Therefore, our results contribute new evidence on the neutral impact of long-term embryo storage on using vitrification for a whole population. The influence of cryopreservation techniques on the stability across storage time is unknown. Has been suggested that the stability of vitrified embryos might be more vulnerable to inherent factors such as changes in pressure or temperature due to alterations in level of liquid nitrogen during filling and maintenance of cryogenic tanks. This could cause crucial effects on molecular mobility and direct molecular damage, causing cracking or fracturing in the vitreous matrix [25,28]. Nevertheless, our results clearly demonstrate that under common daily handling of cryobanks, morulae and blastocysts vitrified using the ministraw device maintained the same viability after a long-term storage period.

## Conclusions

In conclusion, our study showed that a GRB of embryos using vitrification is a successful strategy to re-establish populations in rabbit for at least 15 years. Moreover, our result has important practical implications for the establishment of GRBs in rabbit to ensure that sufficient embryos are available. Our results showed that for this genotype the efficiency was less than 10% in terms of animals that generate offspring. This outcome suggests that increasing the size of the embryo bank could be a strategy to avoid any risk in the future.
