# Correction: Successful re-establishment of a rabbit population from embryos vitrified 15 years ago: The importance of biobanks in livestock conservation

**DOI:** 10.1371/journal.pone.0200403

**Published:** 2018-07-11

**Authors:** Francisco Marco-Jiménez, Manuel Baselga, José Salvador Vicente

There is an error in the affiliation for all authors. The affiliation should be: Institute of Science and Animal Technology, Laboratorio de Biotecnología de la Reproducción, Universitat Politècnica de València, 46022 Valencia, Spain
